# Explaining the facilitators of quality of life in patients with multiple sclerosis: a qualitative study

**DOI:** 10.1186/s12883-021-02213-9

**Published:** 2021-05-11

**Authors:** Atefeh Homayuni, Sedigheh Abedini, Zahra Hosseini, Masoud Etemadifar, Amin Ghanbarnejad

**Affiliations:** 1 Health School, Hormozgan University of Medical Sciences, Bandar Abbas, Iran; 2 Social Determinants in Health Promotion Research Center, Hormozgan Health Institute, Hormozgan University of Medical Sciences, Bandar Abbas, Iran; 3 Tobacco and Health Research Center, Hormozgan University of Medical Sciences, Bandar Abbas, Iran; 4 Neurosciences Research Center, Isfahan University of Medical Sciences, Isfahan, Iran; 5 Department of Public Health, School of Health, Tobacco and Health Research Center, Hormozgan University of Medical Sciences, Bandar Abbas, Iran

**Keywords:** Quality of life, Multiple sclerosis, Qualitative study, Facilitator, Patients

## Abstract

**Background:**

In patients with multiple sclerosis (MS), the disease’s complications and manifestations affect a person’s ability to function normally and leads to further disruptions in their education, family life, job opportunities, and daily life activities, thereby reduce their quality of life. Different factors as facilitators or inhibitors affect the quality of life in patients with MS. This study aimed to explain the facilitators of quality of life in patients with MS.

**Methods:**

This research applied qualitative methodology, utilizing semi-structured interviews with individuals with MS and their family members/caregivers. Purposeful sampling was done among people who referred to Isfahan MS Association. Participants were selected with a maximum variation in terms of gender, age, education, occupation and marital status. Interviews were continued to reach data saturation. The gathered data were concurrently analyzed by the content analysis technique. MAXQDA software version 10 was used for data management.

**Results:**

Saturation was reached after eighteen interviews. A total of three main categories and 8 sub-categories were extracted from the data. The identified facilitators were: personal facilitators (leisure time and coping strategies), interpersonal facilitators (exercise therapy, social support and social organizations) and needs and suggestions for improvement (family therapy, adopting urban architecture and facilities, and supportive systems).

**Conclusions:**

Based on these findings, in order to improve the quality of life in patients with MS, we should pay attention to factors such as leisure time, spirituality and positive thinking, exercise, social support and social organizations. Health professionals, the government, community and families could help to improve patients’ quality of life through adapting urban architecture, holding family therapy sessions and providing supportive systems.

**Supplementary Information:**

The online version contains supplementary material available at 10.1186/s12883-021-02213-9.

## Background

Multiple sclerosis (MS) is an inflammatory and immune-mediated demyelinating disease of the central nervous system (CNS) and the leading cause of non-traumatic neurological disability among young adults [1], affecting women twice or three times as often as men [2], and young individuals aged between 20 and 40 years are mainly affected [3].

According to the results of the study conducted by Azami et al. [4], the prevalence and incidence of MS in Iran is estimated to be 29.3/100,000 and 3.4/100,000. The prevalence of MS in men and women was estimated to be 16.5/100,000 and 44.8/100,000, respectively. The lowest and highest prevalence was found in studies in Southern Khorasan in 2009 (5.3/100,000) and Isfahan in 2013 (89/100,000), respectively. Various factors can play a role in increasing the prevalence of the disease in Iran. These factors include: the lack of particular laws and regulations on the purchase and use of chemicals in Iran and easy access to them, the existence of particles such as PM10 in the air of Iranian cities, natural radiation of radon from soil and unsupervised use of decorative stones and granite in Iran. However, fewer studies have confirmed the relationship between these factors and MS.

Research findings showed that quality of life (QOL) in patients with MS is poorer than for healthy subjects and other chronic diseases, such as epilepsy, diabetes and rheumatoid arthritis [5, 6]. The world Health Organization (WHO) defines QOL as “an individual’s perception of their position in life in the context of the culture and value systems in which they live and in relation to their goals, expectations, standards and concerns” [7].

Overall, there are a number of factors that can significantly reduce or improve the quality of life in people with MS. These factors include disease-related variables as well as a large number of psychosocial factors. Many maintain that the disease itself is not the main factor to poor QOL. Such arguments are consistent with a biopsychosocial model (BPS). According to this model all of the biological, psychological and social factors must be considered simultaneously in order to fully perceive the illness’s impact on patients’ functioning and QOL. Continuation of the disease is one of the factor that has positive and negative effects on QOL [8]. Physical and cognitive disabilities and other neuropsychiatric disorders cause these patients to become dependent on other persons to carry out their daily activities, which can significantly affect their QOL [9]. Perhaps the most severe effect of cognitive impairment in these patients is unemployment, which results in extensive personal, social and financial costs [10]. In addition, the variable and unpredictable course of the disease and its various symptoms including weakness, visual loss, bowel and bladder incontinence, fatigue and mood symptoms can lead to significant psychiatric symptoms and disorders such as depression and anxiety. These psychiatric disorders have been associated with decreased QOL [11–13]. Strober [8] in his study found that individuals with low to average QOL reported worse physical and mental health related quality of life (HRQOL), subjective well-being (depression and anxiety) and psychological well-being. They also experience significantly more stress and less feelings of success in life. They also reported greater fatigue, sleep disturbance and pain. Individuals with high QOL reported higher levels of general and MS-specific self-efficacy and internal locus of control and more perceived social support. These individuals in order to cope with MS use problem-focused and adaptive coping such as planning, active coping, emotional and instrumental social support, humor, acceptance and positive interpretation and growth. It should be noted that in Strober’s study individuals were matched on disease course and duration. Predictors of good QOL in Yamout and his colleagues’s study [5] were: decreased depression score, increased level of social support, religiosity and lower fatigue score. Physical health composite score was increased when social support increased. It was predicted by the disease course (best for RRMS/CIS and worst for PPMS) and decreased by overall EDSS (Expanded Disability Status Scale). And finally, mental health composite score was predicted by fatigue, pain, depression, total education years and social support.

According to what was mentioned above, identification and understanding the influential factors in MS patients’ quality of life can help in effective planning to improve QOL in these patients. Thus, this study aimed to explain the facilitating factors of QOL in patients with MS.

## Methods

### Study design

The present study is conducted with conventional content analysis approach to explore the views and experiences of people with MS about the facilitators of quality of life.

### Study sample and recruitment

Participants were selected with using a purposive sampling and maximum variation (regarding age, gender, education, occupation and marital status). In purposeful sampling, information-rich cases related to the phenomenon are identified and selected. Individuals who can provide the needed information to address research questions [14]. The inclusion criteria of individuals with MS were: 1) having MS diagnosed by a neurologist, 2) having MS for more than 1 year, 3) 3. Having not a chronic disease other than multiple sclerosis, 4) being able to participate in the interview and sharing their experiences and 5) having rich and useful experiences about living with the disease and willing to retell their experiences to the researcher. Individuals were excluded if patients were unable to cooperate and talk due to the worsening of the disease or other reasons and were not willing to continue the interview at the time of the interview.

Some participants were interested in the study, but could not to be interviewed (due to having speech problems, gait disorders and other reasons). So the researcher interviewed with their family caregivers. Some of the criteria for selecting the family caregivers included individuals who were 1) being member of a family as a sibling, parent, spouse or child of patients for more than a year and 2) being responsible for taking care of an individual with MS.

Firstly, the required permissions were obtained from the head of research affairs of the MS Association. In order to enroll the participants in the study, the researcher used different ways such as: an online advertisement posted on the MS Association website, 2) installing an printed advertisement in the MS Association and 3) direct personal interactions with patients in the MS Association. Some explanations about the purpose and importance of the study as well as the conditions for inclusion in the study were offered in advertisements. Individuals who were interested to participate in the study contacted the researcher by telephone and the necessary arrangements were made to interview those who were willing to participate in the study.

The process of data collection and analysis continued until the saturation occurred, a point in the data collection where no new categories emerge [15]. Saturation was reached with 15 participants but we continued interview with 3 additional patients in order to provide greater confidence in the reliability of the study findings. Fifteen people with MS and 3 family members as caregivers were interviewed.

### Data collection

Each participant took part in a semi-structured interview lasting between 30 to 90 min depending on the participants’ interest and tolerance. The interviews were conducted in a convenient time and quiet place for the participants such as the dedicated room in the MS Association or participants’ home.

This interview guide was developed for this study. The guide included two parts: the first part asked for demographic information, including age, age of disease onset, education level, marital status, and so on. The second part included semi-structured open-ended questions which was pilot tested with a scientist experienced in qualitative methods as well as an individual with MS. The primary questions of the interview were:

1. How has this disease affected the various aspects of your life?
2. What are the most important factors that you think affect the quality of your life?
3. What are some of the factors that have contributed to improve your quality of life?
4. What are you currently doing to improve your quality of life?
5. What do you believe are the facilitators to quality of life for patients with MS in the community in the individual, organizational and policy levels? (Caregivers version).

Also, if necessary, we asked in-depth and exploratory questions to elaborate on the details. Examples of these questions were: “Tell me more about it”, “Can you provide an example?”, “If you have a memory about this, tell us”, and so on. All interviews were recorded by audio recorder and transcribed verbatim for data analysis.

### Data analysis

Data collection and analysis were carried out simultaneously. After initial analysis of each interview, the next interview was planned. Each transcript of interview was read several times to identify the concepts hidden in the statements of the participant. After extracting the initial codes, the codes were clustered into categories. Codes that shared similar meanings were grouped together. This process of analysis continued until the emergence of the main and sub-categories. To facilitate the organization and analysis of the qualitative data, the MAXQDA software version 10 was used.

### Rigor

To ensure the trustworthiness of our findings, the criteria suggested by Lincoln and Guba were used [16]. The researcher tried to ensure the credibility of the findings by reviewing the extracted manuscripts and codes, sending the data to colleagues and using their supplementary comments, continuous review and devoting sufficient time for data collection. In the review by the participants, interpretations of the some participants’ explanations were sent to them for review to see whether they are compatible with the participants’ experiences. In continuous review, the researchers used simultaneous data analysis with the data collection process. Sufficient time was also allocated for the interview process. In order to enhance the transferability of findings, samples were selected with maximum diversity. Also, all research processes and characteristics of the study population were described and written in detail by researchers to allow other researchers to follow the research path.

### Ethical considerations

The present research was approved by the Ethics Committee of Hormozgan University of medical sciences (IR.HUMS.REC.1399.065). To ensure voluntary participation in the study, participants were asked to give their consent. All participants were given consent forms to sign. The participants were assured about the confidentiality of the recorded conversations and that they would not be named in the final description and analysis.

## Results

### Participants’ characteristics

The age range of participants was between 29 and 59 years old (mean age = 40.61), and their mean age at the onset of disease was 28.61 years old. Married patients participating in the study had a minimum of 1 and a maximum of 4 children. Four participants had a history of multiple sclerosis in their relatives. All patients received health insurance services.

Other demographic information of the participants is summarized in Table 1.

**Table 1:** Demographic Characteristics of Participants

Variable	N (Percentage)
Gender	
Male	4 (22.2)
Female	14 (77.8)
Marital status	
Single	5 (27.8)
Married	11 (61.1)
Widow	1 (5.55)
Divorced	1 (5.55)
Education level	
Elementary school	2 (11.1)
Junior high school	2 (11.1)
High school and diploma	7 (38.9)
Associate degree	3 (16.7)
Bachelor’s degree	4 (22.2)
Occupation status	
Housewife	13 (72.2)
Employed	3 (16.7)
Unemployed	2 (11.1)
Frequency of hospitalization in the last 1 year	
None	13 (72.2)
Once	4 (22.2)
Twice or more	1 (5.6)
Frequency of the recurrence of disease within the last 1 year	
None	9 (50)
Once	4 (22.2)
Twice	2 (11.1)
More than twice	3 (16.7)
Type of MS	
Relapsing-remitting MS	8 (44.44)
Progressive-relapsing MS	3 (16.67)
Primary-progressive MS	3 (16.67)
Secondary-progressive MS	4 (22.22)

Eventually, data analysis revealed three categories and eight sub-categories. Categories included: 1) personal facilitators, 2) interpersonal facilitators and 3) needs and suggestions for improvement (Table 2).

**Table 2:** Categories and sub-categories extracted of codes obtained from experiences of participants

Category	Sub-category	Repetitive Codes
Personal facilitator	Leisure time	Individual activities/Educational activities/ Social activities/Artistic activities
	Coping strategies	Being positive, Being happy, Giving positive energy to others, Receiving positive energy from others, Believing in destiny, Faith in God, …
Interpersonal facilitator	Exercise therapy	Going to yoga class, Swimming, Aerobic, …
	Social support	Financial support, Mental and emotional support, Physical support, Informational support, ...
	Social organizations	Payment of medical allowance, Financial support, Holding scientific meetings, Arranging for recreational camps, quarterly magazines, Holding educational courses and exercise classes, …
Needs and Suggestions for improvement	Family therapy	Increase families’ knowledge, Adapt to the patient’s disease, Treating the patient,
	Adapting urban architecture and facilities	Audio books, Appropriate transportation, Adapting public places and passages, …
	Supportive systems	Discount on medical expenses, Making medicines available, …

### Category 1. Personal facilitator

This category was the smallest category of the facilitators of MS patients’ quality of life and comprised two sub-categories: leisure time and coping strategies.

#### Leisure time

Planning for leisure time is one of the most important issues in MS patients’ life. Activities such as walking, engaging in appropriate and favorite hobbies, listening to music and reading are important to gain peace and improve physical and psychological functions in patients. Participants stated that their leisure activities included individual, social, artistic and educational activities. Examples of these activities were: reading books, listening to music and audio books, writing, watching sport news, tailoring, going out with friends, talking with others and learning English language.

“My son has been selected for the province’s sport competitions. Sometimes I go to the gym and watch his game, talk to his coach, talk to other athletes. Sometimes I go out with my friends. I have a friend who is a photographer. She says let’s go out, I’m going take photos. I go with her. I entertain myself with trees, flowers and so on”. (Patient, 37-year-old)

#### Coping strategies

In this study, some participants paid special attention to spiritual beliefs and positive thinking in their descriptions. They felt that positive thinking and their spiritual beliefs, as a determining factor, increased their ability to accept the disease and leads to their adaptation to their disease. It also provides a special level of support for patients.

“When I found out that I got this disease, I thanked God. My mother told me whoever gave you the disease, he will keep an eye on you. If God gave you this disease, he would also think of the cure. When I began the therapeutic process, the cost of my medicines decreased by God’s grace. Now, my medicine is nearly free of charge and my family rejoiced at this happening. The first year, my attitude toward God changed significantly” (Patient, 36- year. old)

### Category 2: interpersonal facilitators

Analysis of the interviews content showed that interpersonal facilitators, as one of the facilitators, has an effective role in patients’ quality of life. The sub-categories were exercise therapy, social support and social organizations.

#### Exercise therapy

As a non-invasive and non-pharmaceutical treatment, exercise not only affect the disease components directly but also because of the positive and effective effect on the patients’ psychological and emotional factors, it can have a considerable impact on promoting mental health and consequently coping with the disease and trying to cure it. Some of the participants mentioned the positive effects of participating in physical activities such as improving their physical and psychological functioning.

“From September to the end of December we participated in the Aerobic class which was held in the MS Association. It affected my physical and mental conditions a lot. My physical health improved. Those whose physical health was worse than mine, improved a lot and that was due to going to the Aerobic class (Patient, 43- year. old)

#### Social support

Based on the findings, family and others’ social supports and their appropriate response to MS are among the important factors that affected the patients’ quality of life. This support is provided in the form of emotional and mental assistance, instrumental support, informational and financial support by others especially family members during the treatment period. The majority of participants acknowledged that the presence and providing emotional support by parent, family members, spouse and others in difficult and challenging conditions after the disease’s diagnosis has been a great assistance in accepting and coping with the disease. Without their help and support, it would have been impossible.

“My husband was truly understanding. He supported me a lot. He told me there is no special problem. He supported me mentally. He sympathized with me and took care of me. He didn’t let me suffer and be tired. He even did most of the housework so that I didn’t get tired” (Patient, 39- year. old)

Peers were widely mentioned as a main source of social support. Participants stated that membership in groups of friends who are in a similar situation and receiving their emotional support creates the conditions to better understand each other and cope with the disease.

“I love only my friends. We are all alike. We find true happiness with each other. When I ask my friend ‘Are you fine?’ I understand what I mean by being fine” (Patient, 33- year. old)

#### Social organizations

Participants in the study suggested that social organizations such as charities and associations for supporting patients with special diseases (e.g. MS) play a valuable and effective role in improving their quality of life. Their support, financial or non-financial, involve items such as payment of medical allowance, financial support for families and patients, holding scientific meetings, arranging for recreational camps and quarterly magazines to increase patients’ awareness. Other influential measures are: holding educational courses, exercise classes, counseling and psychology classes.

“I thank God that there is such a thing as MS association. It is a public association which is supported by the government. They provide us with the medication, give us a device for self-infusion, so that you don’t bother to much, give us a bag to carry medicine because all their medicine needs to be stored in cold. It is very useful while travelling. They hold regular conferences and publish books which are truly helpful” (Patient’s wife, 36- year. old).

### Category 3: needs and suggestions for improvement

Among the main categories in this study were needs and suggestions for improving the quality of life in patients with MS. The sub-categories included the adapting urban architecture and facilities, supportive systems and holding family therapy sessions.

#### Adapting urban architecture and facilities

The existing problems in urban design and architecture are one of the biggest barriers to the active participation of disabled or diseased patients (including patients with MS) in social activities as well as enjoying leisure time. The disabled, though enjoying many skills and capabilities, due to these obstacles encounter with problems in accessing to urban environment facilities and thus are inactive. The society is deprived of their potential capabilities, too. Some participants suggested that the urban environment must be adapted to the physical needs of people with physical and mobility disabilities, which bring them back to the society, life and activity.

“The government should design comfortable and suitable facilities for disabled people. In the main streets, in order to easy transportation, certain places can be designed” (Patient’s mother, 35- year. Old).

#### Supportive systems

Most participants stated that economic problems are one of the most important factors affecting the quality of life in MS patients. Economic problems prevent the strict adherence to the diet or the provision of required medicines. Some of the participants stated that the government can increase the supportive systems and take appropriate measures to supply the required drugs at minimum cost or free. By reducing treatment costs, the government play an important role in resolving economic problems related to patients’ treatment.

“Now I want to visit an occupational therapist, I should pay 25,000 bucks per session. Well, we need some discount, I mean more discount. To buy a multi-vitamin syrup, we need to pay 80,000 bucks. That is too much for me, who cannot work and earn money. At least they can lower the price and, thus, support us more. I am not saying that we won’t pay at all. I mean more discount is all we want” (patient, 33- year. Old).

Some other participants complained about the unavailability of medicines especially due to the political and economic sanctions. They found it urgent for authorities to take measures to avail the required medication with no stress for patients at the nearest drugstores to where they live.

“For all of us, the lack of medicine is a problem right now. If only they find a way to reduce the stress related to the lack of the medicine! The rest is up to the patient. It depends on how patient get along with the disease” (Patient’s spouse, 36- year. Old).

#### Family therapy

According to the majority of participants in the present study, the role of family in the process of acceptance, treatment and adaptation to the disease is very important. Families show certain behaviors and feedbacks depending on their internal emotional abilities. Families with effective communication among their members can be more successful and better at helping and supporting their patients. Some participants believed that MS is not an individual disease but a family disease. Thus, families need to be involved too. They need to receive the required training and knowledge too.

“I am eager to spend my leisure time with my family. I like to travel or even stay here in my hometown Isfahan with my husband and sons. Something that never happened. But, they never liked it. I travelled on my own, with my friends, with my mother and sister. I would like to be in company with my family, but they do not wish to” (patient, 43- year. Old)

## Discussion

The present research aimed to explain the facilitators of quality of life in patients with multiple sclerosis. The data analysis revealed 3 main categories (personal facilitators, interpersonal facilitators and needs and suggestions for improvement) as well as 8 sub-categories. In the following, each of these categories and sub-categories is discussed based on the previous studies.

One of the activities that is adversely affected by the disease among MS patients is their leisure time. Leisure activities in these patients are severely reduced and loses its priority. Leisure activities can significantly influence their therapeutic process and improve their quality of life and health [17]. Leisure activities in these patients are simply ignored because they focus on therapeutic measures to alleviate the symptoms and disabilities. However, participating in leisure activities may help to reduce these symptoms or disabilities and consequently reduce the burden of the disease on patients’ families and the wider community [18]. The participants’ experience in this study showed that these individuals spend their leisure time individually or in groups. The literature is indicative of different classifications of leisure activities. For instance, Passmore et al. [19] suggested 3 dimensions (achievement including sports, playing musical instruments, painting or dancing; social including conversation, visiting friends or calling them; and time-out including listening to music or watching TV). Hosseini et al. [18] considered 6 dimensions (including physical, social, individual, artistic/cultural, educational and spiritual/religious activities) of leisure activities.

Among the effective strategies for acceptance and adaptation to the disease which used by the participants were faith in God, surrender to divine destiny and assuming the existing conditions as a kind of divine test. These strategies gave patients some comfort. Faith in God’s will as a superior force reflects the attitude of Iranian women in encountering with the disease and is rooted in Islamic culture and religious teachings. This belief plays a key role in the formation of their health control behaviors, adaptation to the disease, improving their quality of life and mental health. Evidence is indicative of the importance of spiritual factors in the lives of people with MS and other chronic neurological disorders. Research has shown that spirituality has a positive relationship with effective coping strategies and increasing quality of life [20–23]. In their research on MS patients, Brook et al. identified spiritual beliefs and faith as effective factors involved in disease adaptation. Spiritual well-being exerts an appreciable influence on psychosocial adaptation to MS [24]. Moreover, some participants considered the positive effect of features such as optimism, positive thinking and hopefulness in the disease acceptance process and believed that enjoying such features would facilitate the process of adapting to the disease. Studies show that optimism and positive beliefs in general are significantly and positively correlated with different aspects of health, and they play a key role in preventing the spread of physical disorders and increasing hope. Saeedi et al. [25] conducted a study aimed to investigate the effectiveness of positive psychological interventions with Islamic approach to increase hope in females with MS. Their results showed a significant difference between the control and intervention groups in terms of hope.

The findings revealed that participation in sports activities is accompanied by positive effects for patients such as improving physical and psychological functions. As pharmacotherapy has high costs and side effects on MS patients, non-pharmacological methods such as exercise therapy can be an effective and suitable alternative. Today, participation in physical activities are increasingly recommended for managing symptoms, recovering functions, optimizing the quality of life, promoting well-being and increasing participation in daily activities of MS patients. Exercise is one of the best rehabilitation methods for solving multi-faceted problems of MS disease [26]. Numerous studies conducted in Iran and around the world [27–32] revealed that different sports such as aerobics and endurance exercises, yoga, hydrotherapy and so on with different protocols (frequency, intensity, duration and type of activity) has the potential to reduce different physical and psychological problems in MS patients. Pittion et al. [33] reported that reducing the exercise-induced fatigue improves the quality of life in MS patients. This is explained by the effect of physical exercises on the neuromuscular system.

It is evident that patient require support once be diagnosed with the disease. In all conversations with participants, they talked about having a supporter. In different conditions, the type and manner of support is different. These supports include emotional, psychological, instrumental, informational and financial supports on the part of family and friends, groups and private and/or non-private social organizations. Different studies [34–36] have investigated the relationship between social support and quality of life in MS patients. Costa and Calheiros [37] examined the effect of social support on the quality of life in patients with MS. Their results revealed that social support is a predictor with a significant effect on health related quality of life in MS. In their study, psychological support has a more extensive effect than material support. Schwartz and Frohner [38] examined the contribution of medical and social support variables in predicting the mental health dimension of quality of life (MHD/QOL) among patients with MS. The results showed that social support made a significant and unique contribution to the MHD/QOL beyond all the other variables. People with MS who perceived having more social support reported a higher level of mental health. Perceived social support can prevent the adverse physiological effects of the disease. It increases the rate of self-care and positively affect the patients’ physical, social and psychological status and clearly increase the patients’ performance and improves their quality of life [39]. Overall, social support increases adaptation to the disease, quality of life, longevity and outcomes of professional care. It improves economic status, maintain a sense of social homogeneity, facilitates self-evaluation and coping with loneliness [40]. In general, MS patients, due to their undesirable physical conditions, evaluate themselves more negatively and have less hope for the future. Providing social support by family, friends, relatives and social networks can not only contribute to their physical welfare, but can also affect these patients emotionally and lead to an improvement in their quality of life.

Participants in this study also provided some suggestions. Participants’ suggestions for improving the quality of life include adapting urban architecture and facilities, supportive systems, and holding family therapy sessions. Some participants requested an increase in facilities, which are designed and provided according to their needs including designing audiobooks, increasing the services provided by MS association such as caring for patients’ children while the educational and sports classes were held. On the other hand, MS causes disorders and limitations in physical function. As the former life (before the disease) of these patients has been accompanied by peace and comfort and now they suffer from physical disabilities, this disability can reduce their satisfaction with life. On one hand, due to physical limitations, on the other hand, due to the necessity of mental characteristics, these patients require specialized environments that are appropriate for these conditions. Adapting the environment for these patients, reforming the environment and providing the required equipment, help patients to live a peaceful social and urban life, use services and facilities (including public places, passages and urban environment) freely and without the feeling to be in danger, and enjoy their individual independence. Thus, buildings and urban areas need to be designed or adapted in such a way that will compensate patients for their motor limitations. The transportation, access and autonomous use of all people from all urban resources should be provided. Except for safety, security or possession limits, no motor or access limits should be set for these patients, and they should be able to freely transport to these areas and act within them.

Financial problems, paying for treatment, medicine and living costs were among the most important concerns of patients and their families. Some participants appealed to the government support to cover medical, medicine and living costs. The government and charities can play a major role in supporting these patients financially. On the other hand, some participants were dissatisfied with the lack of easy access to medicine, which was stressful to them. Considering the essentiality of patients’ quick access to special medication and in order to facilitating the access to medical services, extensive national plans can be made and coordinated among organizations such as the Ministry of health, health insurance company and NGOs such as MS Support Association and the Charity Foundation for Special Disease to provide patients with the medication they need at the closest drugstore.

Finally, some participants believed that the families should be involved too. Therefore, it is necessary for families to receive the necessary knowledge. As the family is the first and foremost source of care and support for the patient, the family attitude towards the disease, its symptoms and adverse effects can tremendously affect the care provided to patients. Therefore, one of the important care policies is that the family, as a client, needs to know more about the disease, its process and therapeutic procedure. Thus, in order to improve the quality of life in patients and increase adaptation to the disease by them, it is necessary to hold counseling sessions with the patient and family members. During these family therapy programs, it is necessary to pay attention to issues such as teaching how to take care of the patient physically, mentally and psychologically, how family members can adapt to the patient’s disease, as well as how to treat the patient.

### Strengths and limitations

There were some limitations in this study. Since the present study was conducted in Isfahan and this city has particular social, cultural and economic conditions, generalizing the results should be done with caution. Another limitation of this study was the difficulty in recruiting accessing enough number of MS patients from both sexes. Because of the nature of MS disorder, generally the number of men with MS was 2–3 times less than women patient; therefore, balancing the number of male and female MS patients in this study was not possible. Despite these possible limitations, the present study had one strong point, including the fact that we used the experiences of patients who were a member of MS Association along with patients who did not benefit from the services of the MS Association.

## Conclusions

The present findings show that MS patients’ understanding of the facilitators of quality of life included different dimensions such as leisure time, the use of coping strategies, exercise therapy, social support and social organizations. It is certain that awareness of these facilitators can help the family, healthcare system, supportive organizations, the government and society to increase the quality of supportive, educational and clinical services and ultimately improve the quality of life. Therefore, the design and implementation of interventions based on the results of this study can help to increase the quality of life in these patients.

## Supplementary Information

**Additional file 1.**

## Data Availability

The datasets used analyzed during the current study are available from the corresponding author on reasonable request.

## Abbreviations

BPS: Biopsychosocial model
CNS: Central nervous system
EDSS: Expanded Disability Status Scale
MS: Multiple Sclerosis
NGOs: Non Governmental Organizations
QOL: Quality of life
WHO: World Health Organization
