# Supplementary material for: Explaining the facilitators of quality of life in patients with multiple sclerosis: a qualitative study
Source: BMC Neurol. 2021 May 11;21:193. doi: 10.1186/s12883-021-02213-9 (PMC8111999; doi:10.1186/s12883-021-02213-9)
Supplement: Supplementary file 1 — Additional file 1. [file 12883_2021_2213_MOESM1_ESM.docx]

**Interview Guide**

This interview guide was developed for this study. The primary questions of the interview were:

1. How has this disease affected the various aspects of your life?
2. What are the most important factors that you think affect the quality of your life?
3. What are some of the factors that have contributed to improve your quality of life?
4. What are you currently doing to improve your quality of life?
5. What do you believe are the facilitators to quality of life for patients with MS in the community in the individual, organizational and policy levels? (Caregivers version).

When required, follow-up questions were used too, such as

- “Can you explain more?”,
- “Can you provide an example?”,
- “Can you recite any relevant memory?”,
- “What do you mean by saying …?”,
- “How did you feel about …..?”, and
- …….
